# Romantic Love Is Associated with Enhanced Inhibitory Control in an Emotional Stop-Signal Task

**DOI:** 10.3389/fpsyg.2016.01574

**Published:** 2016-10-25

**Authors:** Sensen Song, Zhiling Zou, Hongwen Song, Yongming Wang, Federico d’Oleire Uquillas, Huijun Wang, Hong Chen

**Affiliations:** ^1^Faculty of Psychology, Southwest UniversityChongqing, China; ^2^School of Humanities and Social Science, University of Science and Technology of ChinaAnhui, China; ^3^Sino-Danish College, University of Chinese Academy of SciencesBeijing, China; ^4^Department of Neurology – Massachusetts General Hospital, Harvard Medical SchoolBoston, MA, USA

**Keywords:** romantic love, inhibitory control, emotional stop signal task, sadness

## Abstract

**Purpose:** This study explored whether romantic lovers differ in emotion-related inhibitory control capacity from those who are single.

**Methods:** 88 healthy undergraduate college students participated in the study. Half were currently in love and in a romantic relationship (love group, LG), and half were single and had never been in a romantic relationship (single group, SG). Based on duration of romantic relationship (i.e., love duration), the LG were further divided into two subgroups: “early stage love” and “longer periods of love”. All participants completed an emotional Stop Signal Task, consisting of a variety of human face stimuli displaying either sad or neutral affect.

**Results:** Results found that relative to SG, lovers showed greater inhibitory control [shorter stop-signal reaction time (SSRT)] during negative emotion condition trials. Furthermore, in early stages of love, SSRT for negative emotion condition trials was significantly shorter compared to that in “longer periods of love” or SG individuals, with no significant differences between the two latter groups.

**Conclusion:** Compared with individuals who were single, early stage lovers showed greater capacity for inhibiting action during presentation of negative emotional stimuli. Within a greater social context, greater inhibitory control capacity during early stages of love may be related to the successful formation of romantic relationships, particularly to the ability to persevere in goal-directed action despite negative emotional contexts such as that of sadness.

## Introduction

Romantic love manifests from an integration of behaviors, cognitions, and emotions associated with the desire to enter or maintain an intimate relationship with a specific other person (Aron and Aron, 1991; Cacioppo et al., 2012; Diamond and Dickenson, 2012). This involves behavioral, emotional, and cognitive components (Sternberg, 1986; Hazan and Shaver, 1987). Romantic love is highly correlated with relationship intimacy satisfaction, relationship quality and stability (Riehl-Emde et al., 2003; Acevedo and Aron, 2009), and is even a precondition for marriage (Simpson et al., 1986). Within a wider context, the ability to form meaningful and enduring relationships is an important social skill.

Cognitive control, also known as self-control or executive function (Baumeister and Vohs, 2003), is a skill commonly associated with higher order processing, necessary for goal-oriented behavior. Individual differences in cognitive control capacity have been shown to predict a wide range of behaviors, including forgiveness and faithfulness in close relationships, ability to resist from flirting behaviors with a confederate, and mastery over the desire to meet an attractive person (Pronk et al., 2010, 2011). These findings suggest that the formation of romantic relationships may benefit from optimal cognitive control abilities.

Negative emotions can disrupt cognitive control capacity, such as the ability to inhibit action via top-down mechanisms (Goldstein et al., 2007; Verbruggen and De Houwer, 2007; Kalanthroff et al., 2013; Rebetez et al., 2015). On the other hand, romantic love has been shown to provide resiliency against the adverse impact that negative emotion can have on individuals. For example, a study found that autonomic reactivity (indexed by Respiratory Sinus Arrhythmia) in single individuals decreased during the presentation of negative emotions, indicating a physiological stress response (Schneiderman et al., 2011). No such decrease was found amongst new lovers who began a romantic relationship 2.5 months prior to the experiment, pointing towards enhanced vagal regulation during periods of falling in love (Schneiderman et al., 2011). Furthermore, Studies have even demonstrated pain relief from simply viewing pictures of a romantic partner (Nilakantan et al., 2014). Given the social and biophysiological implications, it is thus important to examine the interaction between emotion and cognitive control within the context of romantic love.

Sadness is a subjective experience that may arise from unpleasant situations and inner feelings of loss or lack of expected gains (Ellsworth and Smith, 1988). In fact, it is reported as one of the most widespread forms of emotional distress. Generally, sadness is thought to motivate vigilant and detail-oriented processing of information, possibly in order to reestablish a sense of control over a situation (Schwarz and Clore, 1996; Bodenhausen et al., 2000; Gasper, 2004). Importantly, although many may differentiate sadness from other negative emotions, to some extent, sadness itself is a negative stimulus that influences attention and results in many similar effects as other negative emotions (Joormann and Gotlib, 2007).

The stop signal task is canonically a behavioral task that is used to examine the ability to control and inhibit ongoing action, called “response inhibition” (Logan and Cowan, 1984; Aron and Poldrack, 2006). In this task, stimuli are presented in regular succession for speeded discrimination responses, but they occasionally appear with unpredictable stop signals that require withholding responses to the target in mid-action (i.e., require inhibition of prepotent motor action). A measure of the stop signal task, the stop signal reaction time (SSRT) is one of the most often used indices of inhibition, where the shorter the SSRT, the greater the hypothesized inhibitory control (Logan and Cowan, 1984). Similarly, the *emotional* stop signal task (eSST) has emerged as a good paradigm for studying the interaction between emotion and inhibitory control that also provides the SSRT metric for analysis and interpretation.

The eSST is an emotion-focused version of the stop-signal task that helps assess how task-related or task-independent emotional signals modulate inhibitory motor control during performance of a behavior (Hare et al., 2005; Pawliczek et al., 2013). In the eSST, emotional pictures are intermixed with neutral stimuli/non-emotional pictures. Interestingly, emotion recognition and emotion regulation are important components of the quality of inter-communication experienced between partners of a romantic relationship (Roisman, 2007). In fact, it has been found that they can help predict marital dissatisfaction and even divorce (Levenson and Gottman, 1985; Gottman and Levenson, 1992). Various studies have also found that negative emotional stimuli can harm inhibitory control capacity, with longer SSRT seen for negative emotion condition trials (Verbruggen and De Houwer, 2007; Kalanthroff et al., 2013). This “negativity effect”, is thought to be attributed to the greater attention and deeper processing that is allocated to threatening information/stimuli (Fox et al., 2002; Vuilleumier and Huang, 2009). In neuropsychiatric disease, negative emotional stimuli have been shown to disrupt inhibitory function in conditions such as anxiety (Sehlmeyer et al., 2010), depression (Gotlib and McCann, 1984; Johnstone et al., 2007; Joormann and Gotlib, 2010) and post-traumatic stress disorder (Frewen and Lanius, 2006).

In the current study, an eSST that incorporates sadness as the emotional experimental stimulus was used to explore the interaction between emotion and response inhibition in romantic lovers, relative to not-in-love single individuals. Interestingly, this kind of interaction may be shown in varying degrees depending on the stage of love one is in, as different stages of love are hypothesized to have different underlying psychological and physiological characteristics. The first phase of an intimate relationship is “being in love” (i.e., early stage of love), and it is characterized by increased commitment, high passion, and a rapid rise in intimacy (García, 1998). This stage plays a key role in the successful formation of pair bonding, and can last from half a year to a year (Marazziti and Canale, 2004). The general features of early stage love may include an altered mood and mental state, as well as excitation and stress caused from insecurity (Stárka, 2007; Berscheid, 2010). These characteristics in new lovers may lead to changes in biomechanisms (Marazziti and Canale, 2004), and may elicit the need for greater cognitive effort when dealing with the risk of a breakdown in a new and still fragile relationship. After several months up to about a year (Marazziti and Canale, 2004; Stárka, 2007), the initial phase of excitation, euphoria and stress, evolves into a “longer period of love”, driven by feelings of balance, safety, and tranquility (Stárka, 2007). It is also composed of features related to passionate love, but in contrast to early stage love, here commitment and intimacy increase steadily and gain more importance (García, 1998). Thus, it is suggested that early stage love may ameliorate the interference of negative emotions on inhibitory control ability, leading to greater inhibition even during emotionally negative conditions, a process theorized to help form romantic relationships. Correspondingly, once having entered later stages of love, decreased stress (Esch and Stefano, 2005), safety and calmness (Stárka, 2007), as well as a relatively stable relationship, may cause a psychophysiological shift where greater inhibitory control capacity becomes less of a necessity.

To better understand this phenomenon and stratify emotional inhibitory control capacity as a function of duration of love, in the current study we employed an emotional stop-signal task. We first ran an exploratory analysis to determine whether there is a significant difference in inhibitory control for emotional condition trials between love and control groups. Then, we performed further analyses to explore possible differences between early stage (ELG) and longer periods of love (LLG). We hypothesized that relative to a control group of single individuals, early stage lovers would demonstrate greater inhibitory control capacity (shorter SSRT), particularly for negative emotion condition trials. On the other hand, an enhanced inhibitory effect was not predicted for romantic lovers in LLG.

## Materials and Methods

### Ethics Statement

This study was approved by the review board and Ethics Committee of Southwest University. Written informed consent was obtained from all participants. All participants were informed that their participation was completely voluntary and that they may withdraw from the study at any time. All participants were over 18 years of age.

### Participants

Eighty-eight students from Southwest University (SWU, Chongqing, China) participated in the study for monetary compensation. They were interviewed at the beginning of the study procedure regarding previous romantic relationships and demographic characteristics. All participants were divided into two groups according to their previous romantic relationship: (1) an “in-love” group (LG; *N* = 44, 21 males) consisting of individuals currently intensely in love [duration of love ranged from 1 to 18 months (8.8 ± 5.0)] – all LG were not married and had no children; and (2) a “single” group (SG; *N* = 44, 19 males), consisting of individuals who had never been in a romantic relationship with anyone before. Based on the distribution of the duration of love, we divided the LG into two subgroups: early stage love (ELG, from 1 to 8 months, *N* = 23), and longer periods of love group (LLG, from 9 to 18 months, *N* = 20).

Age of participants ranged from 18 to 25 years old – LG (21.39 ± 2.1) and SG (21.45 ± 2.2). All participants had normal or corrected-to-normal vision, were right-handed, had no history of attention deficit or learning disabilities, and all were naive as to the purpose of the experiment. Five participants were excluded from further analyses due to high error rate on no-stop-signal trials [more than 3 standard deviations (SD) from the mean], leaving the sample with 83 participants in total for analyses: LG (*N* = 43, 21 males) and SG (*N* = 40, 15 males).

### Questionnaires

#### Passionate Love Scale

The Passionate Love Scale (PLS) (Hatfield and Sprecher, 1986) was used to measure the status of passionate/romantic love in the LG. The PLS has been previously used in samples of Chinese college students (Yin et al., 2013; Song et al., 2015).

### Equipment

Participants sat in a soundproof experiment room with their eyes approximately 100 cm from a 17-in monitor. The screen resolution was 72 pixels per inch, and the viewing angle was 5.7 × 4.6°. A keyboard was placed on the table between the participant and the screen, and participants were tested one by one. All experiment programs were compiled and executed using E-Prime software (Schneider et al., 2002).

### Stimuli

Stimuli consisted of face pictures from the Chinese Affective Picture System (Bai et al., 2006) of males and females displaying either sad or neutral affect. Each emotion category had 40 pictures, half of them males, and the remaining half females. The picture system provided rated valance and arousal values for each stimulus. For valence rating [mean: sad = 3.11 (*SD* = 0.63), neutral = 4.33 (*SD* = 0.45)], sad face pictures were rated more negatively relative to neutral pictures [*t*_(78)_ = -9.96, *p* < 0.001]; for arousal rating [mean: sad = 5.37 (*SD* = 1.34), neutral = 4 (*SD* = 1.12)], sad face pictures were rated as more arousing relative to neutral pictures [*t*_(78)_ = 4.95, *p* < 0.001]. All stimuli were similar to the others in size, background, brightness, contrast grade, spatial frequency, and other physical properties.

### Emotional Stop Signal Task

We used an emotional version of the stop-signal task (Sagaspe et al., 2011). The task included 20 practice trials, which were not further analyzed. Participants were told that the practice trials would be identical to the experimental blocks, except that the experimental blocks would be longer, and would not include feedback.

**FIGURE 1:**
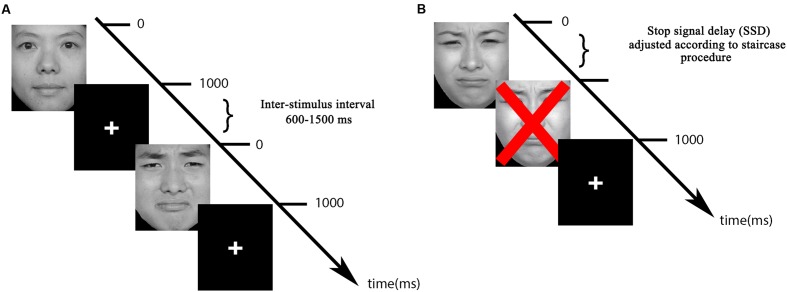
**Task trials depicting one neutral, one sad Go trial (A), and one sad Stop trial (B)**.

Each Go trial began with a fixation cross (the duration was jittered from 600 to 1500 ms), immediately followed by a face stimulus for 1000 ms. Participants were required to discriminate the emotion of the face and answer by pressing the correct keyboard button (for sad emotion: using their right index finger to press “1”; for neutral emotion: using their right middle finger to press “2”; experimental keyboard button rules were counterbalanced) as quickly and as accurately as possible during the 1000 ms presentation (**Figure 1A**). All participants were right-handed and used their right hand to respond.

In Stop trials, a red ‘x’ mark (‘×’) appeared after a face picture and participants were instructed to inhibit their response if they saw the “×” picture after a face picture (**Figure 1B**). The interval between the face onset and the “×” picture (the stop signal delay; SSD) was adjusted online as a function of the subject’s performance on their previous stop-trial with the same facial expression. SSD was initially set at 250 ms. Each time a participant failed to inhibit their response in presence of a stop signal, the SSD decreased by 50 ms. On the other hand, when inhibition was successful, the SSD increased by 50 ms. Thus, stopping difficulty was kept under experimental control on a trial-by-trial basis, eventually obtaining a probability of successful stopping of 50% for both neutral and emotional trials (Levitt, 1971).

Taken together, there was only a 25% probability chance of a “×” picture appearing after a face picture, and the participants did not know when to restrain their reaction. Because they may be tempted to wait for the emergence of an “×”, we instructed to the participants to press the correct key as fast and as accurately as possible, and we emphasized to not wait for a potential stop signal. After practice, there were a total of six blocks of 80 trials each. Each block comprised of 60 Go trials and 20 Stop trials. Half of the trials displayed sad faces, and the other half displayed neutral faces. The experimental trials were delivered in a pseudorandom order, with a maximum of two Stop trials in a row.

Accuracy on Stop trials as well as mean RT was calculated automatically after each block as feedback for the experimenter to verify compliance with the task. After each block, participants were again reminded that both speed and accuracy in each trial were important.

### Data Analysis

Reaction times (RTs) for correct responses during the 1000 ms presentation of the stimuli are reported. The most important indicator describing stopping efficacy that uses RT and is typically derived from a Stop Signal Task is the SSRT. This metric shows the time taken after a stop signal is presented for inhibition to be completed (Logan, 1994). As some participants may strategically slow down their responses over the course of the experiment in order to make inhibiting easier, we adopted the integration approach (Logan and Cowan, 1984; Boehler et al., 2012) instead of the mean approach (Logan, 1994). By taking deviations from an even ratio of successful and unsuccessful Stop-trials into account, this approach is more robust against variations. Go-trial reaction times (goRTs) were rank-ordered, and the RT value at the percentile that corresponded to the percentage of unsuccessful Stop-trials was determined on a subject-per-subject basis (Boehler et al., 2012). Then, the SSRT was calculated as goRT minus average SSD.

Any goRT less than 300 ms (∼1%) in the current study was removed from the analysis. Repeated-measures ANOVAs were performed, with Emotion condition (neutral vs. sad) modeled as a within-subjects factor, and Group (LG vs. SG) as a between-subjects factor. The correct percentage of Go trials were also recorded, as well as unsuccessful rate of stopping and SSRT. In order to further explore differences in inhibitory control across early stage love (from 1 to 8 months, 23 participants), LLG (from 9 to 18 months, 20 participants), and those in the single group (23 participants randomly selected from all 40 single individuals), SSRTs of sad emotion condition trials were further analyzed using one-way ANOVA with Tukey’s Honest Significant Difference (HSD) as *post hoc* analyses.

## Results

The mean PLS score in LG was 96.7 ± 11.19.

For goRT, percentage of correct Go trials and unsuccessful stopping rate showed no significant main effect or interaction (*F*s < 1) (**Table 1**).

**Table 1:** Behavioral performance on the emotional stop signal task (eSST) for LG and SG individuals.

		LG (*N* = 43)		SG (*N* = 40)		ME (*p*) (group)	ME (*p*) (emotion)	Interaction (*p*)
		Mean	*SD*	Mean	*SD*			
goRT (ms)	Sad	580.10	66.07	575.25	55.61	0.674	0.618	0.868
	Neutral	579.10	60.50	573.29	52.52			
Percentage go success	Sad	0.88	0.08	0.89	0.07	0.704	0.683	0.54
	Neutral	0.89	0.07	0.89	0.07			
Percentage stop error	Sad	0.41	0.05	0.42	0.06	0.559	0.749	0.189
	Neutral	0.41	0.05	0.41	0.05			
SSRT (ms)	Sad	211.16	33.86	228.42	36.55	0.159	**<0.001**	**0.013**
	Neutral	203.93	37.10	207.74	35.72			

*LG, love group; SG, single group; SD, standard deviation; RT, reaction time; SSRT, stop signal reaction time; ME, main effect; ms, milliseconds; group = LG, SG; emotion = sad, neutral. Significant effects are marked in bold*.

**FIGURE 2:**
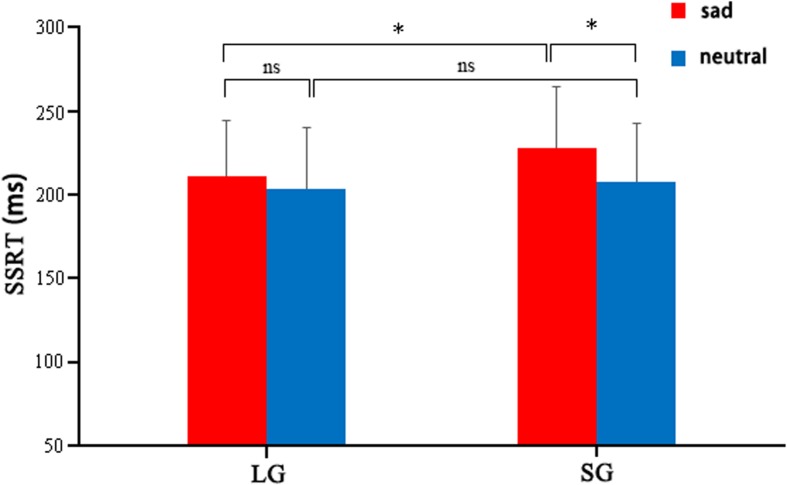
**Emotional Stop Signal Task (eSST) performance between LG and SG, by emotion condition (neutral, sad)**. eSST, emotional Stop Signal Task; SSRT, stop signal reaction time; LG, love group; SG, single group. ^∗^*p* < 0.05; ns, not significant.

For SSRT, there was a significant main effect of emotion condition [*F*_(1,81)_ = 27.731, *p* < 0.001, η^2^ = 0.255], with longer SSRT (indicating poorer response inhibition) in the sad compared to the neutral condition trials. No significant main effect of group was observed [*F*_(1,81)_ = 2.022, *p* > 0.05] (**Table 1**). A significant interaction between emotion condition and group was found [*F*_(1,81)_ = 6.445, *p* = 0.013, η^2^ = 0.074]. A simple main effect analysis showed that the SSRT for sad stimuli was significantly shorter in LG than in SG [*F*_(1,81)_ = 4.988, *p* = 0.028, η^2^ = 0.058], but that SSRT for neutral stimuli was not significantly different between LG and SG [*F*_(1,81)_ = 0.227, *p* > 0.05, η^2^ = 0.003]. Concurrently, while in SG the SSRT for sad stimuli was significantly longer than the SSRT for neutral trials [*F*_(1,81)_ = 29.394, *p* < 0.001, η^2^ = 0.266] (**Figure 2**), in LG SSRT was similar between sad and neutral trials [*F*_(1,81)_ = 3.678, *p* > 0.05, η^2^ = 0.045].

### Differences across Early Stage Love, Longer Periods of Love, and SG for SSRT of Sad Emotion Condition Trials

**FIGURE 3:**
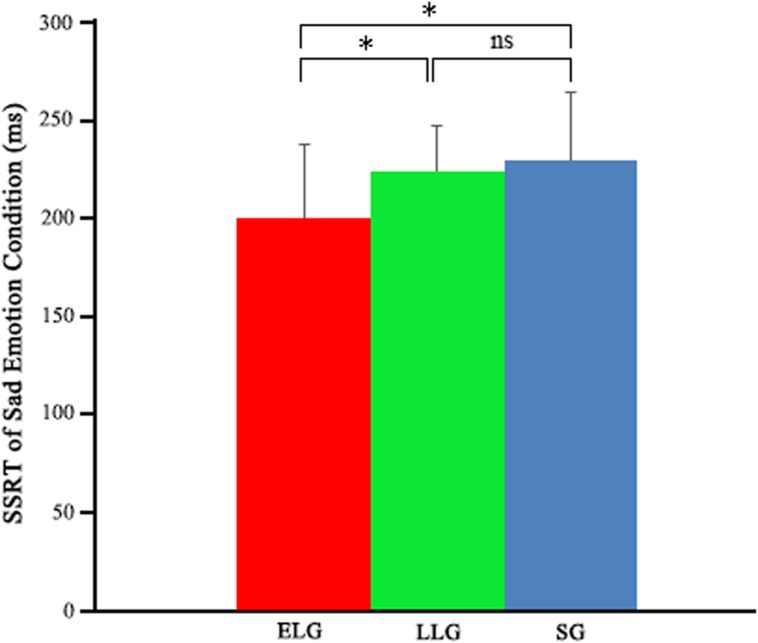
**Emotional Stop Signal Task performance (sad condition) between ELG, LLG, and SG**. eSST, emotional Stop Signal Task; SSRT, stop signal reaction time; ELG, early stage lovers; LLG, longer period lovers; SG, single group. ^∗^*p* < 0.05; ns, not significant.

Significant differences in the SSRT of sad emotion was found across these three groups: early stage love (199.84 ± 37.85), longer period love (224.17 ± 23.29), and SG (228.52 ± 35.09), *F*_(2,65)_ = 4.96, *p* < 0.01. In *post hoc* analyses, significant differences for SSRT of sad condition trials were observed between early stage love compared with both individuals in LLG (*p* = 0.049) and SG (*p* = 0.013), but no significant differences between the LLG and SG were found (*p* > 0.05) (**Figure 3**). Similar results were found when including participants’ PLS scores as a covariate to the group of lovers.

## Discussion

To our knowledge, this is the first study investigating the possible effects of romantic love on negative emotion-related inhibitory control capacity. We found that sad face stimuli in the negative emotion condition of the eSST can interfere with inhibitory control mechanisms, as demonstrated by the longer SSRT relative to the SSRT of the neutral condition. Furthermore, romantic love may attenuate the inhibitory control impairment effect associated with unpleasant emotion priming. Compared to the single group, in lovers, we found enhanced inhibitory control during the presentation of sad stimuli. Furthermore, the lovers in the early stage had better inhibitory control performance for negative emotion condition trials than those in LLG. These findings suggest that individuals in early stages of romantic love could benefit from enhanced self-control ability, allowing for relationships to form and mature.

### The Influence of Sad Emotion on Cognitive Control

We found prolonged SSRT for sad (negative) emotion stimuli trials compared to neutral stimuli trials, consistent with several previous studies using an eSST (Verbruggen and De Houwer, 2007; Herbert and Sütterlin, 2011; Kalanthroff et al., 2013), and a go/no-go task (Goldstein et al., 2007). Essentially, the ability for stopping via top–down control mechanisms was impaired on average during the presentation of a negative stimulus.

Numerous studies suggest that emotionally salient cues (for example, threatening stimuli or angry faces) can intensely impact attention (Lerner et al., 2012; Pourtois et al., 2013; Carretié, 2014), that being the capacity to selectively respond to pertinent aspects of the environment while suppressing potential sources of distraction and competing courses of action (Desimone and Duncan, 1995; Miller and Cohen, 2001). Furthermore, emotion and cognition have been classically viewed as rival forces (Damasio, 2005; Okon-Singer et al., 2007). Specifically, a threatening stimulus consumes processing resources that are needed for successful inhibitory performance (Pessoa, 2009; Pessoa et al., 2012). From these perspectives, threat-related emotional stimuli may disturb cognitive processes.

Although sadness differs from other negative emotions (for example, anger) with lower arousal levels, it is arguably more far-ranging, and longer lasting in daily life relative to other emotional states (Ellsworth and Smith, 1988). To some extent, it is also a negative stimulus that influences our attention (Joormann and Gotlib, 2007). Some early research studies suggest that sadness reduces the efficiency of attention-related tasks, and may constrict the attention range (Potts et al., 1989). Still others have shown sadness to be related to higher physiological impulsivity (Camras and Allison, 1989), as well as a tendency to overly focus on the self and one’s self image (Wang et al., 2005). Several cerebral limbic and paralimbic systems have been identified when coping with sadness, including the ventral medial preFrontal Cortex (vmPFC) (Suslow et al., 2013), Anterior Cingulate Cortex (ACC) (Habel et al., 2005), Insula (Liotti et al., 2000), and Amygdala (Aldhafeeri et al., 2012). However, few studies have examined the specific influence of sadness on response inhibition via an eSST. As the neural mechanisms also remain unclear, these should be addressed in future studies as it was not the main focus of the present study.

### Romantic Love Can Modulate the Influence of Negative Emotion on Inhibitory Control

This study provides the first evidence that romantic love may modulate the interference of negative emotion on inhibitory control. Our results also offer a further extension to earlier work about intimate relationships and self-control. In particular, we demonstrate that individuals in the early stage of love (ELG) group showed greater response control during the sad emotion condition trials. However, relative to ELG, this improvement in inhibitory control was absent in those who were in the LLG.

The disparate results between ELG and LLG are consistent with characteristics of the two stages of love. For example, during the initial stages of love, it is important to efficiently inhibit any harmful or impulsive behavior arising from negative emotion in order to form an intimate relationship with the special partner – a lack of constructive reaction to negative emotions may halt or stand in the way of successful formation of the intimate relationship. Interestingly, early stage lovers also seem to experience significant pain relief when looking at a picture of their partner (Nilakantan et al., 2014), a potentially beneficial survival mechanism that goes hand in hand with enhanced cognitive control mediated by romantic love. Previous studies have found that those in longer partnerships (8–17 months), rather than the early stage (1–8 months), showed greater activity in the ventral pallidum (Aron et al., 2005). The ventral pallidum has been implicated in attachment in prairie voles (Lim and Young, 2004; Lim et al., 2004). Thus, after several months of stressful interplay, the lovers enter into a calm and safe state (García, 1998). The experience of attachment starts to accompany feelings of romantic love (Fisher, 2004; Acevedo et al., 2012) and the commitment aspect of love becomes more important during longer-term stages. By this stage, they have formed a relatively stable close relationship with their partner(s) and the need for greater cognitive control to integrate negative emotions diminishes.

Improved cognitive control in romantic lovers is in general in line with with several studies showing that romantic love can be beneficial for cognitive control (Bianchi-Demicheli et al., 2006; Nilakantan et al., 2014; Wlodarski and Dunbar, 2014). For example, studies including participants from early to longer stages of love (but without the additional analyses differentiating between the two phases), suggest that presentation of a love-related stimulus can also prime love-relevant networks and enhance subsequent performance on conceptually related mentalizing tasks (Wlodarski and Dunbar, 2014). Even the subliminal presentation of a romantic partner’s name, in contrast with a friend’s name, can facilitate cognitive performance on a lexical-decision task (Bianchi-Demicheli et al., 2006; Ortigue et al., 2007).

Furthermore, the potentially enhanced social inhibition capacity in lovers may be related to increased parasympathetic activity as measured by the Respiratory Sinus Arrhythmia response to negative stimuli. This response works to prevent autonomic stress and facilitates emotion regulation in the early stages of love (Schneiderman et al., 2011). Overall, properly adjusting current action in order to make better suitable behavioral responses to lovers, friends or even strangers, is dependent upon the proper recognition of others’ explicit negative demeanor (such as sadness).

The neural mechanisms for the greater cognitive function found in lovers may be related to increased functional connectivity (FC) in frontal areas. Our previous study (Song et al., 2015) for example, found that the FC between the temporo-parietal junction, ventromedial prefrontal cortex, and dorso-medial prefrontal cortex were increased in lovers. These brain networks are known to subserve social cognition. Additionally, some of these regions and networks have been consistently suggested to be associated with ‘theory of mind’ tasks (the ability to determine other people’s emotions and intentions) (Gallagher and Frith, 2003), facial expression recognition (Winston et al., 2002), and have been reported in several functional Magnetic Resonance Imaging (fMRI) studies of lovers (Aron et al., 2005; Xu et al., 2011; Acevedo et al., 2012).

The enhanced cognitive function in lovers may also be the result of neural biochemical mechanisms. Studies show a critical role for oxytocin (OT) in promoting pair-bonding formation, and OT has been shown to modulate social interaction and sexual satiety in prairie voles (Carter, 1992; Williams et al., 1992; Witt et al., 1992). Importantly, significantly elevated plasma OT has been found during the early stages of human romantic relationships (Schneiderman et al., 2012).

Furthermore, in order to solve stressful situations (Marazziti and Canale, 2004), cortisol levels elevate, and testosterone levels and follicle stimulating hormone are down regulated in early romantic love (de Boer et al., 2012). This early phase is also characterized by low serotonin levels (Marazziti et al., 1999) and high nerve growth factor (Emanuele et al., 2006). However, during later stages of love, levels of several neuroendocrine substances found to be changed in early romantic love, return back to normal levels (de Boer et al., 2012). For example, platelet serotonin transporter (Marazziti et al., 1999) and nerve growth factor (Emanuele et al., 2006), return to normal levels, and stress is decreased (Esch and Stefano, 2005). Because of the potential use of these substances as biomarkers for love (including duration of love) and their potential psychophysiological effects, in future studies we aim to collect and explore these biochemicals across early stage and the late stages of love.

On a related note, the current study has some implications for addiction. For example, romantic lovers can show impulsive behaviors that are similarly found in addiction (e.g., obsessive thinking about the person, increased energy, as well as emotional dependency on and craving for emotional union with the beloved) (Aron et al., 2005), and these behaviors are sometimes even viewed as a form of natural reward addiction (Fisher et al., 2016). Furthermore, both romantic love and drug addiction display functional enhancement of reward and emotion regulation networks (Breiter et al., 1997; Aron et al., 2005; Ortigue et al., 2007; Volkow et al., 2007; Frascella et al., 2010; Xu et al., 2011). However, the current findings and our previous fMRI study results suggest that romantic love displays special functional enhancement in social cognition networks (Song et al., 2015), while drug addiction displays special dysfunctions of cognitive control networks (Goldstein and Volkow, 2011). Thus, cognitive control seems to be the key difference between romantic love and addiction. In turn, we see that greater inhibitory capacity in early stages of love may thus not only help form close relationships with others, but also help to develop and mature those relationships in a healthy manner, in contrast with drug and behavioral addiction. Because of these similarities and key differences, a better understanding of how romantic love improves cognitive control during early development of love could help inspire new treatments for drug addiction.

### Limitations and Future Directions

Despite the novel results of the study, there are several limitations to be acknowledged. First, only sad and neutral emotion stimuli were used in the current study, and thus, findings may not be arbitrarily generalized to other negative emotions (e.g., fear and/or anger). Thus, cognitive control capacity in other emotional contexts needs to be explored in future studies. Second, duration of love within the sample only ranged from 1 month to 18 months, and all participants were not married and had no children. Thus, the current results do not allow us to make any assumptions about longer-term durations of romantic love (e.g., several years). Because of this, it is also unknown if the early stage of a new marriage or that of having a child would show similar enhancement of inhibitory control, which could serve as an interesting topic for future research studies. Third, longitudinal studies would be very helpful in disentangling how and why inhibitory control changes across the development and maturation of romantic relationships, perhaps providing more causal findings than what is possible with cross-sectional studies such as the present one.

## Conclusion

To conclude, in the current study we found that early stage lovers, compared to later stage lovers and single individuals, had greater inhibitory control performance during negative emotion condition trials of an eSST. Greater inhibitory control capacity in those who are in love may help form and maintain romantic relationships in the fragile initial stages. These results shed light on the possible benefits of being “in love” on cognitive control and inhibition capacity, and demonstrate the possibility of applying an approach without using love-relevant cues (e.g., pictures of romantic partners) for investigating the influence of romantic love on cognition.

## Author Contributions

SS is responsible for the original experimental design, data analysis and article writing. ZZ is responsible for the experimental process, data collection, and article writing. HS is responsible for experimental design and data analysis. YW is responsible for behavioral questionnaire data and experimental procedure plan. FdU is responsible for manuscript writing, copy editing, and content editing. HW is responsible for chart and graph arrangement, including **Table 1** and **Figures 1** to **3**, arrangement of data, and proofreading of the manuscript. HC is responsible for experimental design and guidance throughout.

## Conflict of Interest Statement

The authors declare that the research was conducted in the absence of any commercial or financial relationships that could be construed as a potential conflict of interest.
